# Asymptomatic Effect of Occupational Radiation Exposure on Thyroid Gland Hormones and Thyroid Gland Ultrasonographic Abnormalities

**DOI:** 10.3390/jcm7040072

**Published:** 2018-04-09

**Authors:** Khaled Alawneh, Musa Alshehabat, Haytham Al-Ewaidat, Liqaa Raffee, Duaa Forihat, Yousef Khader

**Affiliations:** 1 Department of Radiology, Faculty of Medicine, Jordan University of Science and Technology, Irbid 22110, Jordan; 2 Department of Veterinary Clinical Sciences, Faculty of Veterinary Medicine, Jordan University of Science and Technology, Irbid 22110, Jordan; maalshehabat@just.edu.jo; 3 Faculty of Applied Medical Sciences, Allied Medical Sciences, Jordan University of Science and Technology, Irbid 22110, Jordan; haewaidat@just.edu.jo; 4 Department of Accident and Emergency, Faculty of Medicine, Jordan University of Science and Technology, Irbid 22110, Jordan; laraffee5@just.edu.jo; 5 Dental Medical Center, Jordan University of Science and Technology, Irbid 22110, Jordan; dnffeihat@just.edu.jo; 6 Department of Public Health and Community Medicine, Faculty of Medicine, Jordan University of Science and Technology, Irbid 22110, Jordan; yskhader@just.edu.jo

**Keywords:** occupational radiation exposure, radiation, thyroid function, T_3_, T_4_, TSH

## Abstract

Data evaluating the effect of asymptomatic effects of radiation on thyroid hormone panels and ultrasonographic abnormalities among radiology technologists are scarce. This study aimed to determine the asymptomatic effect of radiation in a total of 39 male and 11 female exposed radiology technologists working in radiology departments, and a total of 34 male and 16 female age-matched controls working in other departments in the same hospital. The level of triiodothyronine (T_3_), thyroxine (T_4_) and thyroid-stimulating hormone (TSH) were evaluated using Enzyme Linked Immunosorbent Assay (ELISA). Thyroid ultrasonographic evaluation outcomes were given as normal or abnormal. There was significant interaction between exposure and gender in the mean TSH and T_4_ but not T_3_. The mean TSH for exposed men was significantly higher than that among non-exposed men (2.28 mIU/L vs. 1.59 mIU/L; *p*-value = 0.003). The mean TSH was not significantly different between exposed and non-exposed women. The mean T_4_ for exposed men was significantly higher when compared with non-exposed men (11.1 pmol/L vs. 10.05 pmol/L; *p*-value = 0.005). In the non-exposed group, 93.8% of women and 94.1% of men had T_4_ values lower than established normal reference range, while in the exposed group, 90.9% of women and 74.4% of men had low values of T_4_. There was no significant difference in mean T_3_ between exposed and non-exposed groups for men and women. Furthermore, there were no significant differences in the thyroid gland ultrasonographic findings between exposed and non-exposed groups. Occupational radiation exposure is associated with increased means of TSH and T_3_, especially among men.

## 1. Introduction

Radiation in physics is defined as the emission or transmission of energy in the form of waves or particles through space or through a material medium including humans and animal models. Radiation can be classified into ionizing and non-ionizing energy, based on the damaging effect on living tissues. A common source of ionizing radiation includes X-rays, which are globally used at medical facilities and research centers [1]. A high dose of ionizing radiations is well documented to cause detrimental effect on humans including cancer induction of various tissues [2]. Lower doses of radiation may potentially increase risk of health hazards in particular for chronically exposed or improbably protected workers [3]. 

Thyroid gland function is controlled by the hypothalamic pituitary thyroid axis, which maintains the thyroid hormones, including triiodothyronine (T3) and thyroxine (T4). Thyroid hormones are regulated by the thyroid-stimulating hormone (TSH) produced by the anterior pituitary, which itself is regulated by the thyrotropin-releasing hormone produced by the hypothalamus [4]. Thyroid hormones have strong influences on energy metabolism and temperature regulation. Thyroid diseases such as hypothyroidism, thyroiditis and cancer can potentially causes mental, physical and even death if not properly treated [4]. Direct or incidental thyroid gland exposure to radiation may disturb the function of the thyroid gland.

There is debate concerning the potential risk and impact of low-dose radiation for occupationally exposed humans. Most studies have evaluated the effect of short-term but high-dose radiation exposure, and only limited studies have evaluated the effect of long-term and low-dose radiation exposure in occupationally exposed workers [5]. In one study, occupational radiation was positively associated with breast cancer risk [6]. Only very few studies have evaluated the effect of low-dose radiation exposure on the level of thyroid hormones. In one animal study, there were no significant variations in concentration of T3 and T4 in the blood of the rats that had been irradiated at doses of 0.5, 1, 2, 4 and 6 Gy when compared with non-irradiated rats [7]. Meanwhile, other animal studies have suggested that long-term low-radiation exposure (0.5 Gy) seemed to inhibit biosynthesis of T3 and T4 in rats [8]. 

The International Commission on Radiation Protection and the National Council Protection and Measurements has established an annual exposure limit and protective guidelines against over-exposure. However, our observation in daily practice, thyroid protection shields are taken lightly and are frequently not taken seriously by the medical personnel. Thus, it is intuitive to assume that the thyroid is relatively more exposed to damaging ionizing radiation among persons who are generally exposed to radiation. Therefore, the main aim of the study was to assess the level of the thyroid hormone panel as well ultrasonographic abnormalities of the thyroid gland in a randomly selected group of occupationally exposed radiology technologists working at the radiology departments in one university hospital.

## 2. Experimental Section

This study was comparative cross-sectional in design. It involved one group of all working radiology technologists at the radiology departments, and one group of age-matched randomly selected non-exposed groups working at other departments in the same hospital. All enrolled participants had signed a written informed consent prior to participation following approval from the Institutional Review Board (IRB). The exposed group were included in the study if their present occupation involved routine and regular exposure to radiation. The non-exposed control group were included in the study only if they had no history of radiation exposure and worked in the same hospital. Participants from either group who had a history or confirmed diagnosis of thyroid cancer, hypothyroidism, hyperthyroidism or thyroiditis were excluded from the study. Pregnant women were excluded from the study as well. Blood samples were collected to evaluate the thyroid hormone panel (T3, T4, TSH) and thyroid ultrasonographic evaluation was performed by blinded board-certified ultrasonographic specialist. The analysis of the thyroid hormone panel was performed using Enzyme Linked Immunosorbent Assay (ELISA) according to the manufacturer’s instructions. The ultrasonographic evaluation of the thyroid gland was categorized as normal or abnormal based on the finding of nodules or cysts.

### Statistical Analysis

All statistical analyses were performed using SPSS software (SPSS version 19.0, SPSS Inc., Chicago, IL, USA). The quantitative variables were expressed by the mean and standard deviation while qualitative variables were expressed in absolute frequencies and percentages. Differences in the characteristics between the two groups were evaluated using the Student’s *t*-test for continuous variables and Pearson chi-squared test for categorical variables. Differences between groups were considered statistically significance if the *p* value was ≤0.05.

## 3. Results

This study included a total of 50 (39 men and 11 women) radiology technologists and 50 (34 men and 16 women) age-matched non-exposed health professionals. There was significant interaction between exposure and gender in the mean TSH (*p*-value = 0.009) and T4 (*p*-value = 0.045) but not in T3 (*p*-value = 0.608). This indicates that the effect of exposure on TSH and T4 differed according to gender. 

Table 1 shows the findings of the thyroid hormone panel and the thyroid ultrasonographic findings among exposed and control groups. The difference in the mean level of TSH between exposed group and control group was significant among men only. The mean TSH for exposed men was significantly higher than that among non-exposed men (2.28 mIU/L vs. 1.59 mIU/L; *p*-value = 0.003). In the non-exposed group, only two men showed higher values of TSH and only one women showed lower value of TSH when compared with the our laboratory established reference normal values of TSH. Meanwhile, in the exposed group, only one woman showed higher values and one men showed lower values of TSH. Moreover, the effect of exposure was significant among men only. 

The mean T4 for exposed men was significantly higher than that among non-exposed men (11.1 pmol/L vs. 10.05 pmol/L; *p*-value = 0.005). In the non-exposed group, 93.8% of women and 94.1% of men had T4 values lower than normal values while in the exposed group, 90.9% of women and 74.4% of men had low values of T4. 

No significant difference was observed in the mean of T3 between exposed and non-exposed groups for men and women. Furthermore, there were no significant differences in the thyroid gland ultrasonographic findings between exposed and non-exposed groups.

## 4. Discussion

Risk of health complications among radiology technologists occupationally exposed to low and chronic doses of radiation is considered to be one of the most important issues in occupational health. The current study was undertaken to explore whether exposed radiology technologists working at radiology departments at one referral university hospital are at higher risk of asymptomatic thyroid hormones and gland abnormalities. The main findings of the research presented here indicate that there is a significant interaction between radiation exposure and gender with regard to mean TSH and T4, but not T3. In a study that evaluated the effect of different doses radiation on the concentration of T3 and T4 in blood of non-irradiated and irradiated rats suggested that the effect of radiation may be mediated by the anorexia syndrome associated with radiation [7]. A recent study suggested that occupationally exposed radiographers in a known area to have iodine deficiency have increased risk of thyroid nodules [9]. Furthermore, a study reported by Chen et al. have suggested that thyroid disease among physicians may be significantly associated with other risk factors including heavy work stress and working night shifts [4]. The study reported here represents a small-sized cross-sectional design. An increase in the TSH and T4 simultaneously may also suggest a secondary rather than a primary process in the thyroid gland. Authors speculate with regard to several factors, including autoimmune mechanisms, and gender distribution in this study, which mainly consisted of men. Furthermore, in this study, hormones were only and randomly measured once, and thus it is hard to make a definitive conclusion as the circadian secretion of thyroid hormones is well documented. The findings may also suggest the transient effect of radiation on the thyroid gland and thus studies with more sample size and homogenous gender distribution that document multiple readings of hormones are needed. 

Thyroid nodules or tumors have been suggested to occur more frequently in occupationally exposed people to radiation. Studies have suggested that exposure to short-term and high-dose ionizing radiation may increase the incidence rate of thyroid cancers. The number of children with thyroid cancer has markedly increased in the former Soviet Republic of Belarus, near the Chernobyl reactor, from one case per million children under the age of 15 per year to about 80 per million children per year in the worst-affected area [10]. In our study, although thyroid nodules were more frequent in the exposed group, there was no significant association between thyroid ultrasonographic abnormalities and the status of exposure to radiation. There have been many risk factors associated with thyroid goiter or nodules including iodine deficiency, gender, alcohol intake, pregnancy status and using oral contraceptives [11]. In a study reported by Trerotoli et al., it was suggested that occupational exposure to radiation combined with mild iodine deficiency was not a significant risk factor for developing nodular lesions in the thyroid glands [12]. Our findings are in agreement with previously reported research efforts. It has been reported that thyroid nodules are common and are commonly benign where the prevalence depends on the studied population and method of evaluation [13]. Additionally, it has been reported that the incidence of thyroid nodules increases with age and is more commonly reported among women when compared with men. The most commonly used sensitive and cost-effective imaging modality to detect thyroid nodules is the ultrasound, with incidental thyroid nodules are being more frequently discovered, which may suggest the benign nature of thyroid nodules in many cases as thyroid tumors are extremely rare in the non-exposed adult population [10,13,14].

Limitations of the current study include the small sample size evaluated over a short period of time in only one referral center. Furthermore, the authors did not record data on the dose of the medical radiation exposure. In conclusion, the findings of the current study provide supporting evidence that exposure to radiation may be associated with increased thyroid gland hormones. Further studies with larger sample sizes, including more health centers, and considering iodine status, are thus required. In this study, one major limitation is the gender distribution where males represented most of the enrolled participants and thus larger study with homogenous gender distribution and evaluating the age or medication history as a cofounder is needed. Based on the findings, professionals who work on a regular basis in an occupation that involve radiation ought to follow radiation protection recommendations including workers’ education about radiation safety, radiation dose monitoring and reporting and applying all protective shielding devices.

## Figures and Tables

**Table 1:** The differences in the means of thyroid gland hormones between radiology technologists (exposed to radiation) and non-exposed health professionals.

Gender	Exposed Group		Non-Exposed Group		*p*-Value
	*n*	Mean ± SD	*n*	Mean ± SD	
Thyroid-stimulating hormone (mIU/L)					
All	50	2.12 ± 1.11	50	1.74 ± 0.94	0.076
Women	11	1.53 ± 0.49	16	2.06 ± 1.30	0.212
Men	39	2.28 ± 1.18	34	1.59 ± 0.69	0.003
Thyroxine (pmol/L)					
All	50	10.88 ± 1.57	50	10.18 ± 1.44	0.021
Women	11	10.10 ± 1.38	16	10.43 ± 1.29	0.540
Men	39	11.10 ± 1.55	34	10.05 ± 1.50	0.005
Triiodothyronine (pmol/L)					
All	50	5.04 ± 0.46	50	4.92 ± 0.52	0.201
Women	11	4.71 ± 0.28	16	4.55 ± 0.54	0.334
Men	39	5.14 ± 0.46	34	5.08 ± 0.42	0.626
Abnormal thyroid gland ultrasonographic findings, *n* (%)					
All	50	16 (32)	50	13 (26)	0.509
Women	16	8 (72.7)	16	7 (43.8)	0.137
Men	34	8 (20.5)	34	6 (17.6)	0.756
